# A Bayesian Scene-Prior-Based Deep Network Model for Face Verification

**DOI:** 10.3390/s18061906

**Published:** 2018-06-11

**Authors:** Huafeng Wang, Wenfeng Song, Wanquan Liu, Ning Song, Yuehai Wang, Haixia Pan

**Affiliations:** 1 Department of Electronics and Information Engineering, North China University of Technology, Beijing 100144, China; wangyuehai@ncut.edu.cn; 2 Department of Software, Beihang University, Beijing 100191, China; swfbuaa@163.com; 3 Department of Computing, Curtin University, Perth, WA 6102, Australia

**Keywords:** Bayesian network, deep learning network, scene transfer, deep features, face verification

## Abstract

Face recognition/verification has received great attention in both theory and application for the past two decades. Deep learning has been considered as a very powerful tool for improving the performance of face recognition/verification recently. With large labeled training datasets, the features obtained from deep learning networks can achieve higher accuracy in comparison with shallow networks. However, many reported face recognition/verification approaches rely heavily on the large size and complete representative of the training set, and most of them tend to suffer serious performance drop or even fail to work if fewer training samples per person are available. Hence, the small number of training samples may cause the deep features to vary greatly. We aim to solve this critical problem in this paper. Inspired by recent research in scene domain transfer, for a given face image, a new series of possible scenarios about this face can be deduced from the scene semantics extracted from other face individuals in a face dataset. We believe that the “scene” or background in an image, that is, samples with more different scenes for a given person, may determine the intrinsic features among the faces of the same individual. In order to validate this belief, we propose a Bayesian scene-prior-based deep learning model in this paper with the aim to extract important features from background scenes. By learning a scene model on the basis of a labeled face dataset via the Bayesian idea, the proposed method transforms a face image into new face images by referring to the given face with the learnt scene dictionary. Because the new derived faces may have similar scenes to the input face, the face-verification performance can be improved without having background variance, while the number of training samples is significantly reduced. Experiments conducted on the Labeled Faces in the Wild (LFW) dataset view #2 subset illustrated that this model can increase the verification accuracy to 99.2% by means of scenes’ transfer learning (99.12% in literature with an unsupervised protocol). Meanwhile, our model can achieve 94.3% accuracy for the YouTube Faces database (DB) (93.2% in literature with an unsupervised protocol).

## 1. Introduction

**Figure 1:**
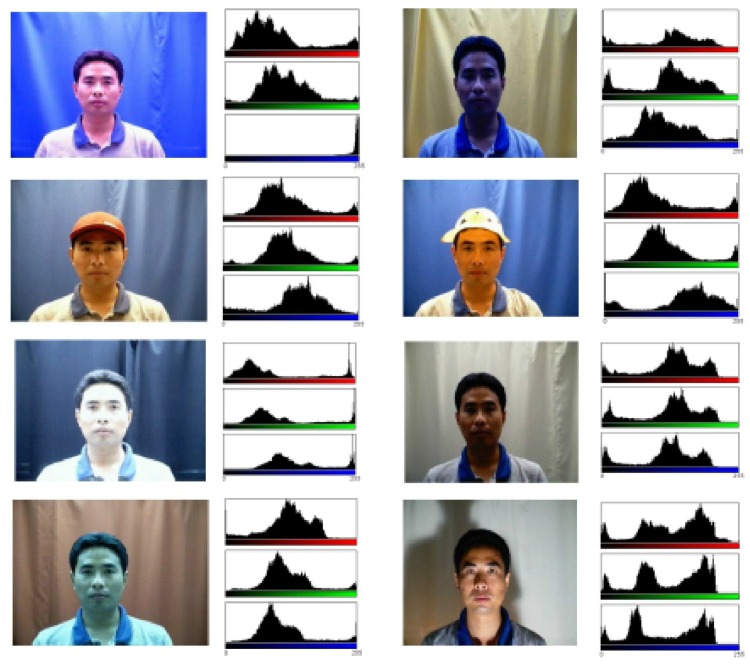
Faces in various illuminated scenes: the histograms indicate the large varieties for the same individual in different scenes.

Face verification or recognition has attracted much attention with rapid progress in the past two decades, particularly equipped with recent deep learning techniques for significant performance improvement. However, its high performance usually depends on features extracted from a large number of labeled training samples as requested by the developed deep learning techniques. In these large samples, one main challenge is to recognize a number of faces captured in various scenes that do not appear in such training scenes. Several previous works [1,2,3,4] have already suggested that an acquired face should be regarded as a mixture of two components, one being information in the the face region, such as pose [5], expression [6], and age [7], and the other being the backgrounds associated with the face region, illumination [8], and so on, with an example shown in Figure 1. We note that we do not deal with pose or expression separately as other researchers have done before; we integrate them into background scenes and tackle them in one framework.

Although most of the previous works in literature perform very well for popular face databases such as the LFW and YTF datasets [9,10], they still to some extent rely on the background variance of the dataset, which is referred to as the “scene” in this paper. That is to say, they require the training dataset to be large enough, that is, including many samples, to represent sufficient scenes as requested. Currently, many researchers focus on proposing approaches for face recognition/verification on the basis of idealistic scenes and validate their methods on specific datasets while ignoring the fact that the face samples in training and testing sets are frequently present in different scenarios. Therefore, many previous approaches are limited or fail for some applications if the size of the training samples is quite limited in terms of scenes. In order to build a robust face recognition or verification system, effective feature extraction as well as semantic scenes extracted from training samples are highly recommended [11]. The extraction of scene semantics in natural scenes has been well studied in [12] and [13]; we borrow the scene concept for face semantic segmentation tasks. In brief, the motivation of this paper is attributed to the fact that the same person with various backgrounds can improve the face-verification performance. This rationality can be justified by the following two aspects: on one hand, the dataset is effectively augmented via domain transfer learning; on the other hand, the final features learned from deep neural networks (NNs) are facilitated because of the enrichment of individual face scenes used for training. In summary, the main contributions of this paper can be summarized as follows:

- We propose a scene model based on the Bayesian deep network technique, which can infer several complicated scenes for the face-verification task.
- A new unsupervised face-verification model is developed on the basis of the scene transfer learning technique.
- Experiments on two challenging datasets validated the proposed model in the case of a lack of sufficient training samples.

The organization of the rest of the paper is as follows: In Section 2, we review the literature in this area; Section 3.1 develops the Bayesian prior scene model, and Section 3.2 focuses on the scene inference. Finally, in Section 4, we propose the deep learning model and present some quantitative results of our Bayesian scene based network model for face verification. The conclusions are given in Section 5.

## 2. Previous Work

**Figure 2:**
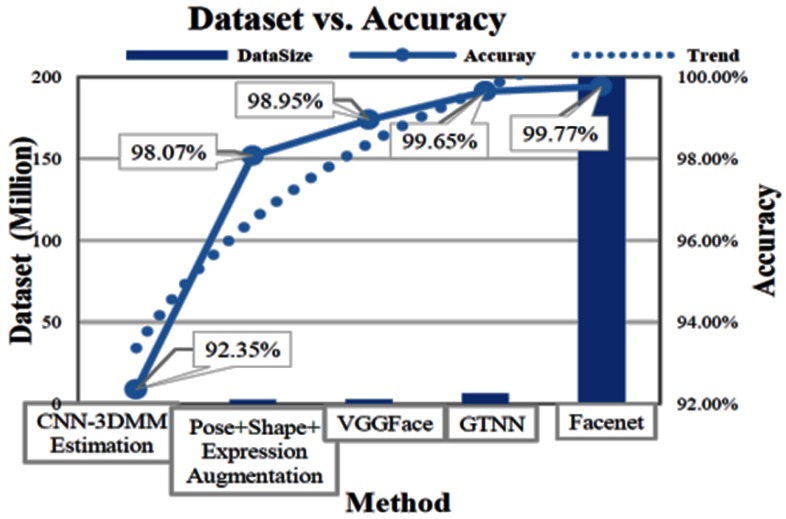
An illustration of accuracy with increasing dataset size.

As deep learning NNs are our main concern in this paper, we only review some results related to NNs. In literature, there are three main categories of networks related to the proposed network model: highly deep network based (HDNB), large-dataset-based (LDB), and multimodal-based (MMB) networks. As for the HDNB network, this category needs to use labeled data with a very deep network for training in order to achieve higher accuracy. In practice, the HDNB approaches rely on a deep structure, and they comprise a long sequence of convolutional layers. For example, the deep residual net [14] has more than 1000 layers and achieved 5.71% top-1 error on ImageNet validation. In 2014, Christian Szegedy [15] proposed a 22 layer net, the “Google Net”, which is a network with a carefully crafted design that allows for increasing its depth and width while keeping the computational budget constant. However, this type of network model cannot be too deep because of vanishing gradients. Fortunately, Ropes Kumar Srivastava et al. [16] developed an approach to enable the depth of the net to increase without the vanishing-gradients constraint. They use the “transform gate” to transmit the information derived from the input data to keep the gradient descent from vanishing. Even with hundreds of layers, such networks still can be trained directly through simple gradient descent. Their research enlightened the study on extremely deep architectures with efficiency. LDB methods aim to train a classifier with a large dataset. Yana Taiga et al. [14] used 4 million data samples with a bootstrapping process and improved the transferring capability of the network with the aim of discovering the connection between the representation and the capability of discrimination. In 2015, Google [17] proposed a convolutional neural network (CNN) model (FaceNet) that could directly learn a compact Euclidean mapping from face images. As reported, it could achieve a highaccuracy of 99.6% on the LFW benchmark [10]. However, this approach needs to be trained on a large dataset with a data size of about 200 million. Yana Taigman [18] (Deepface) employed a 3D face model to align using locally connected layers without weight sharing. Later they declared [3] that the CNN method has a bottleneck with increasing data. They proposed a solution for alleviating this by replacing the naive random subsampling in the training set with a bootstrapping process. Moreover, a link between the representation norm and the capability of discrimination in a target domain was discovered, and this research sheds lights on how such networks can represent faces. Although it is suggested that the larger the data size is, the higher accuracy one can achieve, it is also clear that the last 0.4% point is hardly to be achieved by only increasing the size of the training dataset. Particularly, 0.1% improvement needs an increase in the data size of 199.99 million; the tendency is shown in Figure 2. MMB approaches are in essence associated with ensemble methods, which involve multi-models to work together. Jintao Liu et al. [16] (the Baidu Face) exploited a two-stage method based on multi-patch features and metric learning with triplet loss. Their method achieved 99.77% for pairwise verification. However, it tends to deplete too many computing sources because it needs too many overlapped computing jobs. Sun Jain et al. [19] tried different structures and different patches to extract the aligned face features. After that, they proposed a classifier to differentiate between different faces (verification or identification) and achieved high accuracy on both the LFW benchmark and Casia [2] dataset.

As far as above three categories to be concerned, there is a consensus that the key issues to improve the face recognition/verification performance are to design a reasonable deep network with appropriate data size and then to work with a hybrid process model. However, the reality is that we can often have different methods while lacking an appropriate size of labeled data at hand. This is in fact the well-known problem of a small number of training samples, and it has been investigated extensively by the computer vision community [20]. This problem has not been sufficiently tackled for deep learning NNs, and currently researchers are trying hard to develop new network structures or promote broad applications for the currently existing network structures. This is mainly due to the fact that deep learning NNs are very complicated, as they aim to mimic the human brain functions and are still in a developing stage. To our surprise, the networks for the small data size problem do not perform as well as human beings. That is to say, human beings can distinguish between a large number of faces by using very few datasets in learning. For example, the work by Salakhutdinov, Ruslan et al. [21] could extract new features from very few training examples by learning both low- and high-level generic features, and these features could fully represent correlations between low- and high-level features. Motivated by their approach, we define the high-level features as a scene in this paper and propagate these scene factors to a deep learning network that is exploited to learn a practical model from input face images. Details are presented in following sections. For convenience, the related symbols and concepts are first listed in Table 1.

## 3. The Proposed Methodology

**Figure 3:**
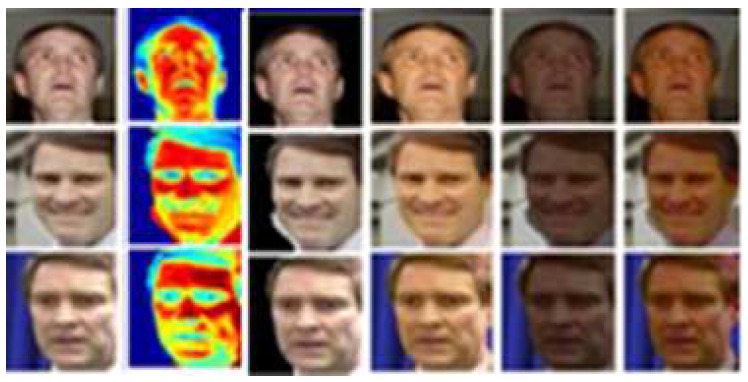
The scene illustration by process of conditional random field (CRF) transferred to our proposed method.

To better understand the method presented in this paper, we need to clarify the concepts of the high-level features or scene first. Previously, S. Zheng et al. introduced a new form of CNN that combines the strengths of CNNs and conditional random field (CRF)-based probabilistic graphical modeling for segmentation [13,22,23]. Motivated by the proposed methods in [13,24], we embed the scene concept into the above semantic segmentation task. By “plugging” CRFs into the CNN, we can obtain a new deep network that has desirable properties derived from CNNs and CRFs. Although the network was originally designed for semantic segmentation, it provides great benefits when we integrate this idea into our approach for scene extraction and scene backwards propagation. In Figure 3, the first column is the original scene, and after the semantic segmentation shown in the second column, we can deduce more new scenes from columns 3 to 6, as we explain in the following sections.

### 3.1. The Bayesian Scene-Prior-Based Deep Network Model

**Figure 4:**
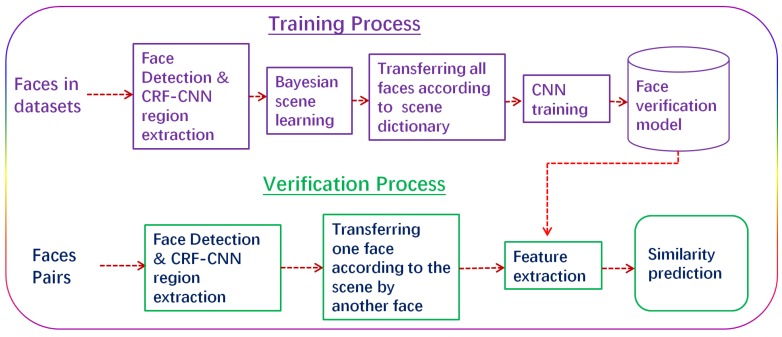
A pipeline for the proposed method.

**Figure 5:**
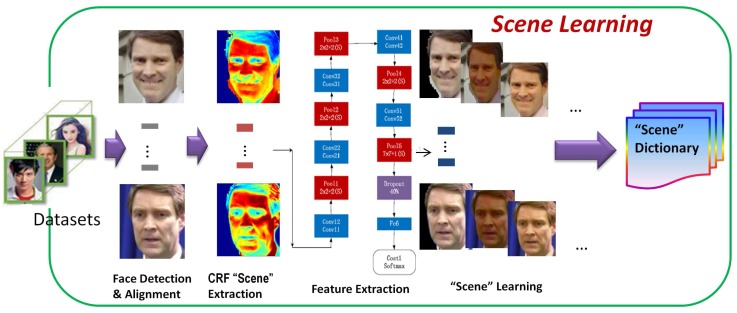
A brief view of the scene learning model.

First, the proposed pipeline for face verification in this paper is outlined in Figure 4. As shown in Figure 4, the whole process consists of two steps: the training and the verification. Next, we explain each part in detail. We first propose a combination of a Bayesian network and a CNN, which concerns both the global and the local feature distributions. Similarly to a human being’s cognitive process, it is believed that a good object classifier should first have a good capability to understand the scenes, and then its capacity for knowing about objects’ existence in these scenes can be much improved. In view of this, the proposed method consists of the following steps: (1) to learn the scenes; (2) to express the scenes; (3) to feed scene factors into the CNN training process and optimize the parameters for face verification; and (4) to finally feedback the learnt knowledge into a new learning iteration step if a new scene is given. Inspired by the contributions of a previous study that utilized the latent Dirichlet allocation (LDA) model to learn the natural scene categories [12], we propose a similar new method to learn the scene distributions between the face pairs’ overlapping spaces. In this context, we consider the scene variance as continuous and use the mixture Gaussian model instead of the multi-nomial model to describe such distributions. The main idea is to transform a face pair into a very close scene in order to lessen the possible scene variation effect in face verification. As shown in Figure 5, after roughly detecting and aligning the face images from a dataset, the detected faces are segmented by the CRF to build a series of scene candidates. These preliminary candidates are then dealt with by the succeeding CNN feature extraction in order to learn a scene expression. Ultimately, a scene dictionary is output according to the distance measurement among any given face pair for the same person.

For clarity, we further define the scene learning by a rigid mathematical description:

(1) $$s=\{s_{1},s_{2},\cdots ,s_{M}\},$$

(2) $$d\left(face_{i},face_{j}\right)=\sqrt{{\left(face_{i}-face_{j}\right)}^{T}\Sigma^{-1}\left(face_{i}-face_{j}\right)},$$

where Σ is the covariance matrix, and *M* is the number of scenes for a given person. The initial dictionary entry is determined on the basis of the distance *d* between the given face pairs.As noted, the covariance matrix is estimated by the face feature vector for the same person.

For the purpose of generating an augmented new dataset according to the learnt scenes, we need to know the relationship between the scenes and features first. Once a scene entry in the dictionary is given, the feature distribution for the same person can be expressed as a mixture Gaussian distribution, as below:

(3) $$p\left(x\right)=N\left(x|\mu ,\Sigma \right)=\frac{1}{{\left(2\pi \right)}^{N/2}}\left|\Sigma \right|exp\left\{-\frac{1}{2}{\left(x-\mu \right)}^{T}\Sigma^{-1}\left(x-\mu \right)\right\}.$$

Given the parameter θ, and where μ and Σ are the distribution parameters’ mean and the covariance to be learnt in the training process, then given the scene *S*, the feature *f*, and the scene *S*, the model described in Equation (4) is followed:

(4) p(f,s)=p(f|s)p(s)→N(θ|s).

**Figure 6:**
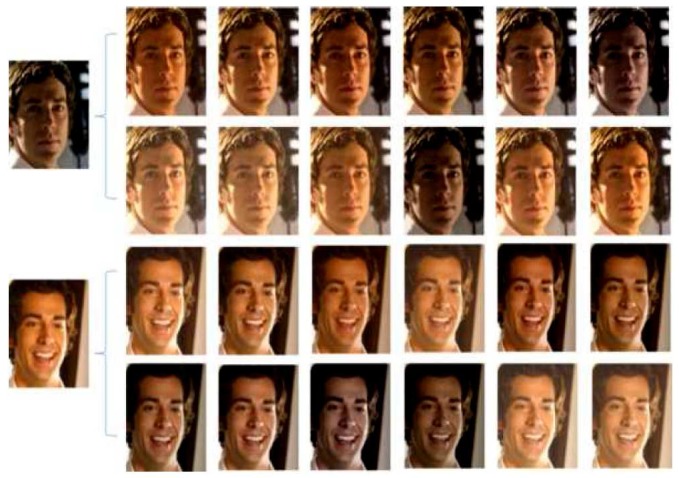
Generated faces according to the scenes.

When any two faces of a pair have a different θ value, the distance between them will be relatively large; conversely, the distance tends to be small. At the beginning of the procedure for our model, the face categories or scene entries *C* are randomly initialized. We suppose that there is a dataset containing *M* images denoted by $X=(x_{1},x_{2},\cdots ,x_{m})$ for a given person *i*. For convenience, we use the symbol *x* instead. One image may have *K* scenes with all the probabilities correlative to the change in the faces. In order to decide which scene a given image belongs to, the nearest-neighbor rule is applied to the set of the probabilities calculated from Equation (4) above. The scenes are not in discrete space, but they are continuous in s∈[0,1]. The closer *s* is to 1, the more likely the image belongs to the given scene. Therefore,

(5) $$p\left(x\right)=\sum_{s}p\left(s\right)p\left(f\;|s\right)=\sum_{k=1}\pi_{k}N\left(x_{k}|\mu_{k},\Sigma_{k}\right),$$

where $\pi_{k}$ is the distribution of scene $s_{k}$. Eventually, more faces can be generated for the imbalance training dataset according to Equation (5) on the basis of the learnt scene dictionary, as shown in Figure 6.

**Figure 7:**
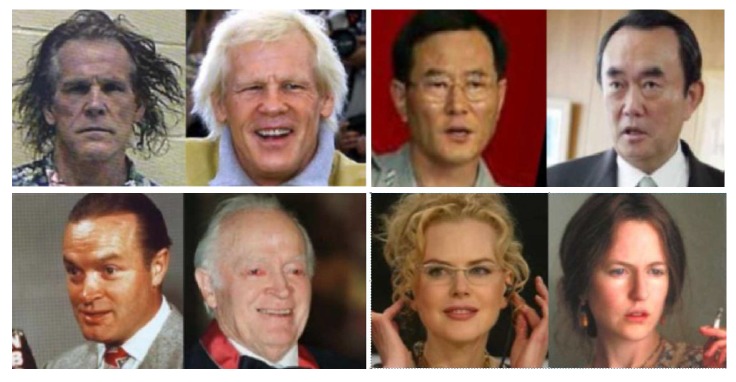
The face pair with different scenes (each image has a different scene).

As for the face pairs shown in Figure 7, Equation (4) can be expressed in another way, as follows:

(6) p(x,y)=p(x|s)p(s|y)p(y).

Furthermore, on the basis of the conditional distribution in the graph model of p(x|s), we denote this latent variable by $\gamma (s_{k})$; thus,

(7) $$\begin{array}{c} \gamma \left(s_{k}\right)=p\left(s_{k}|x\right)=\frac{p\left(s_{k}\right)p\left(x|s_{k}=\gamma_{k}\right)}{\sum_{l=1}^{k}(p\left(s_{k}=\gamma_{k}\right)p\left(x|s_{k}=\gamma_{k}\right)}\\ \;\\ \;\;\;\;\;\;\;\;\;\;\;\;\;\;\;\;\;\;\;\;\;\;\;\;\;\;=\frac{\pi_{k}N\left(x|s_{k},\Sigma_{k}\right)}{\sum_{l=1}^{K}(\pi_{l}N\left(x|\mu_{l},\Sigma_{l}\right)}, \end{array}$$

where $\pi_{k}$ is the prior probability of a scene. The above Bayesian model describes the prior relationships among the images, features, and image categories. An image needs a scene to describe the condition that a face is shown in it; therefore, one person’s face may have several scenes to be assigned to. In this context, the scene model is used as a prior to the image, and then we exploit the semantic pixels to determine the face region. This priori information is backward-propagated to the next iteration for generating more reasonable faces. We usually refer to the scenes as the learnt higher-level features, each of which will have a dramatic effect on the distribution of the face images. During this iteration, the face in different scenes is determined semantically [25] by calculating the low-level (pixel-level) distribution, which can be expressed as a Dirichlet distribution. The scene of a given face is one of *K* random variables subject to

(8) $$0\leq \gamma \left(s_{k}\right)\leq 1,\sum_{k=1}\gamma \left(s_{k}\right)=1.$$

Up to here, we have addressed how to create *K* scenes from training samples; next we explain how to infer a scene for a given face.

### 3.2. Scene Inference

Each image is a mixture of scenes, where the corresponding latent variables $\gamma (s_{k})$ are denoted by an N×K matrix *x* with $s_{n}^{k}$ rows (for a given person with *n* face images and *K* scenes). We let *s* be the *k*th scene, such that

(9) p(s|x,μ,∑)∝p(x|s,μ)p(s|∑)∝p(x|s,μ,Σ),

where μ and Σ are parameters learnt from the ground truth. The PDF of a scene is given by Equation (9). The scene can be considered as a main background factor for the identity of a given object, which is learnt from mixed image spaces. When a new scene is to be learnt, the original imbalanced data will even be augmented by generating new scene images. Eventually, this enlarges the original image space in the direction of the missing data characteristics by using iterative backward propagations. The number of scenes is a latent variable, which is a constant and is determined by the images from the training set. Then, the scene *s* is subject to

(10) $$s=arg\underset{s}{max}\left(p\left(s|\theta \right)\right).$$

Now the problem is how to propagate the scene information to the data features and make the data richer with the new scene feature. The aim is to obtain $p(image|s_{mix})$, where $s_{mix}$ is the hybrid face scene in a verification task. Up to here, we have already been able to calculate p(x), p(c), p(s), p(s|x,c), and p(f). Hence, the goal can be achieved by the following steps:

(1) Perform convolution and pooling on I(u,v).
(2) Determine a mixed feature space $\varphi_{mix}$ or a mixed image space, as shown in Equation (11).
  By using the L2-norm, the mixed image space can be derived by the following equation:
  (11) $$\phi_{mix}=\left\{\left(i_{1},i_{2}\right)|Malanobis\left(f\left(i_{1}\right),f\left(i_{2}\right)\right)\leq \varepsilon \right\},$$
  where $i_{1}$ and $i_{2}$ are two different images; *f* is the feature extracted from the images; and ε is an empirical value initialized within [0.3,0.5], as we need 90% error rate hits when the similarity is in this interval.
(3) Decide p(x|s,θ). The term p(x|s,θ) is in general obtained by integrating over the hidden variables π and *s*.
  (12) $$p\left(x|s,\theta \right)=\int p\left(\pi |\theta \right)(\prod_{n=1}^{N}\sum_{s_{n}}p\left(s_{n}|\pi \right)p\left(x_{n}|s_{n},\theta \right))d\pi$$

As defined previously, θ represents the parameters to be already learnt with θ=(μ, Σ) and $s_{n}$ is the *n*th scene. When a scene is given or the distribution of the scene is fixed, we solve Equation (12) by maximizing the log of likelihood function; thus we have

(13) $$\begin{array}{c} ln\;p\left(x|\pi ,\mu ,\sum \right)=\sum_{n=1}^{N}ln\left\{\sum_{k=1}^{K}\pi_{k}N\left(x_{k}|\mu_{k},\sum \_k\right)\right\}\\ \;\\ \;\;\;\;\;\;\;\;\;\;\;\;\;\;\;\;\;\;\;\;\;\;\;\;=-\frac{ND}{2}\left(ln\;2\pi \right)-\frac{N}{2}ln\;\left|\sum \right|-\frac{1}{2}\sum_{n=1}^{N}{\left(x_{n}-\mu \right)}^{T}\Sigma^{-1}\left(x_{n}-\mu \right), \end{array}\;$$

where μ and Σ are determined by the derivative of the log likelihood with respect to μ. In fact, μ and Σ are estimated by $\overset{\wedge }{\mu },\overset{\wedge }{\Sigma }$, as follows:

(14) $$\overset{\wedge }{\mu }=\frac{1}{N}\sum_{n=1}^{N}x_{n},\hat{\Sigma }=\frac{1}{N}\sum_{n=1}^{N}\left(x_{n}-\overset{\wedge }{\mu }\right){\left(x_{n}-\overset{\wedge }{\mu }\right)}^{T}.$$

Because the parameters *s* and θ are coupled, the variational approximation technique is used to maximize the log likelihood of the data and to minimize the Kullback–Leibler divergence between the approximation and the true posteriors. Here, we use the distribution q(.) to approximate the true distribution. Then we optimize Equation (12) by maximizing the lower bound of the likelihood. The variational lower bound on the marginal likelihood for a single labeled face image can be computed as follows:

(15) $$\begin{array}{c} \log p(x|s,\theta )\\ \;\\ \geq \sum_{s}q\left(\pi |s\right)\log p\left(\pi ,s,x|\theta \right)-\sum_{s}q\left(\pi ,s\right)\log q\left(\pi ,s\right)\\ \;\\ =E_{q}\left[\log p\left(\pi ,s,x|\theta \right)\right]-E_{p}\left[\log xq\left(\pi ,s\right)\right]. \end{array}$$

By defining L(γ,x,θ) for the right-hand side (R.H.S.) of the above equation, we have

**Figure 8:**
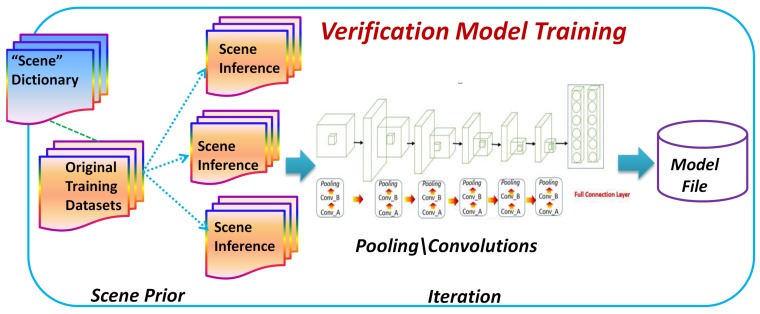
The illustration of the procedure for training a verification model on the basis of scene inference.

(16) logp(x|θ)=L(γ,x,θ)+KL(q(π,s|γ)||p(π,s|x,θ)),

where KL() is the Kullback–Leibler divergence between any two distributions, q(π,s|γ) is an arbitrary variational distribution, and γ is the above-mentioned latent variable. The second term on the R.H.S. of the above formula stands for the KL distance of the two probability densities. As shown in Figure 8, the original size of the training datasets is finally enlarged according to the scene inference procedure, and then the enriched datasets are fed into a model training process in order to obtain a model for the next verification task.

### 3.3. Hyperparameter Optimization

By simply considering the Softmax loss or the Euclidean loss, the loss function is able to be affected by noisy scenes. Thus, we propose a ground truth distribution model based on the mixture Gaussian model. We denote the NN energy by $E_{in}$ and the distribution energy by $E_{scene}$. Then we form the following distribution:

(17) $$\begin{array}{cc} E & =E_{in}+E_{scene}+\zeta \\ & =\frac{1}{2}||f\;\left(I\left(x\right)\right)-{y||}_{2}^{2}+\alpha KL\left(p\left(x|\theta \right),q\left(x|\theta \right)\right), \end{array}$$

where $I\left(x\right)=\left(I_{1},I_{2},\cdots ,I_{N}\right)$ are the input images; ζ is a penalty item, which is a constant here; and α is the coefficient factor. The energy function has two main components. The first component is a plain NN error, which aims to make the predicted result much closer to the ground truth. However, if we only have this term, a large dataset is required in order to achieve a relatively higher accuracy. The training process also tends to be out of control after learning a batch size of the dataset and then results in an overfitting. As we know that the CNN was originally designed to learn the general features in a class, it cannot fit the variance of the training images. Thus we add the second term to generate the variance for the existing images, and we enlarge the variance by generating different scenes for a given image via the scenes’ transformation. The bound becomes tight if and only if p(x)=q(x). In addition to maximizing the log likelihood of the dataset, the conditional constraint (as shown in Equation (17)) could also select parameters that minimize the Kullback–Leibler divergence between the approximation and true posteriors. For implementation, the energy is approximated by an expected maximum (EM)-based variational learning method.

### 3.4. Overlapping Distributions’ Transform

The validation dataset falls into two parts: one is data to be recognized easily with relatively discriminative feature spaces; the other contains some of the less familiar images that lie in a crossing space and that cannot be directly classified. We name all such image datasets in the crossing space as $\phi_{mix}$; thus,

(18) $$\begin{array}{c} \phi \left(p_{k},q_{k}\right)=\left\{\left(x_{i},x_{j}\right)\right|KL\left(p\left(s_{k}|x_{i)},1\left(s_{k}|x_{j}\right)\geq \eta \right)\right\}\\ \;\\ \;\;\;\;\;\;\;\;\;\;\;\;\;\;\;=\{\left(x_{i},x_{j}\right)|Malanobis(s_{k}|x_{i},s_{k}|x_{j})\geq \varepsilon )\} \end{array}$$

(19) $$\phi_{mix}=\overset{K}{\underset{k=1}{⋃}}\phi \left(p_{k},q_{k}\right),$$

where η or ε is the threshold to determine the marginal of the overlapping space. Before we have the samples (refer to Section 3.1) to be generated for the next iteration and take the scene as a prior for the next time, we treat the difference in the pair as the scene variance. Then we can enlarge the distance in the overlapping classes’ space with the dataset *s* and the scenes in *s*. Finally, we obtain the easily wrongly labeled faces and scenes for the overlapping space. However the difficulty is that the selected features are not sufficient to discriminate between the current varying scenes. Luckily, we are motivated by the human visual system in which, when people cannot recognize one person by the given low-level features, they would try to find more discriminative high-level features. Usually they extract the high-level features or semantic features. Similarly, we use the face region generated by the CRF to extract high-level semantic features for the recognition task.

**Figure 9:**
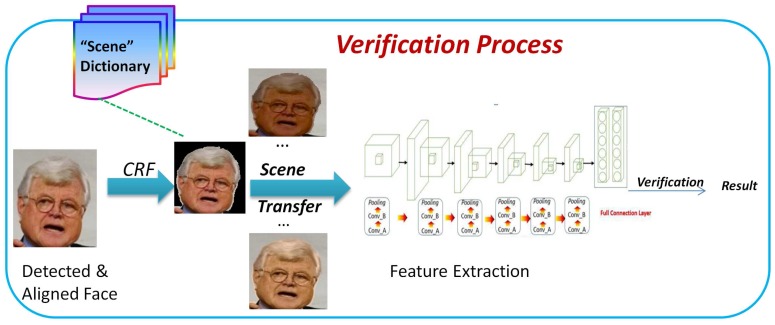
The semantic features’ extraction procedure.

The semantic feature extraction procedure is as shown in Figure 9. In this procedure, a semantic feature is determined by the CNN extracted feature on the basis of the face region produced by the CRF. As shown in Figure 9, the extracted features will be used as input for subsequent face-verification tasks. We note that the main purpose for the scene dictionary exploited in this context is to help the transformation of a given face into a specified scene.

### 3.5. Scene Backward Propagation

Now we describe how to obtain the scene distribution among the images in the training dataset. As for Equation (17), the gradient of $E_{scene}$ can be expressed as follows:

(20) $$\begin{array}{c} \frac{\partial E_{scene}}{\partial s_{k}}=\frac{\partial E_{scene}}{\partial f_{l}}\frac{\partial f_{l}}{\partial s_{k}}=\delta ,\\ \frac{\partial E_{scene}}{\partial \theta }=\frac{\partial E_{scene}}{\partial f_{l}}\frac{\partial f_{l}}{\partial s_{k}}\frac{\partial s_{k}}{\partial \theta }, \end{array}$$

(21) $$=\delta \Delta p\left(x|s_{k}\right)=\delta \prod_{k=1}^{K},N\left(\Delta \mu_{k},\Delta \Sigma_{k}\right),$$

where *l* represents the layer in the NN, θ is a parameter of the scene distribution, and $f_{l}$ is the output activation function. The scene is propagated by the overlapping space. Then we can extract the scene distribution in Equation (22) and transform it back to the first layer of the NN.

(22) $$p\left(x^{t+1}\right)=p\left(x^{t}\right)+\delta \prod_{k=1}^{K}N\left(\Delta \mu_{k},\Delta \Sigma_{k}\right),$$

where $\mu_{k}$ is the learning rate for scene propagation, and δ is the gradient of a scene in pairs. The whole process is listed in the algorithms in the appendix.

## 4. Experiments and Results

For face detection, a face detector is used on each image, and a tight bounding box around each face is generated. These face thumbnails are resized and aligned to a size of 141×165 pixels. At the beginning, we use a CNN model to train the deep features. The training- and validation-related topics are given in the following sub-sections.

### 4.1. Datasets and Evaluation

The new method was evaluated for a face-verification task; that is, given a pair of two face images, a squared Cosine metric $\tau (x_{i},x_{j})$ was used to determine whether the two images were the same or a different person. We used CASIAWebFace [25], which contains 10,575 subjects and 494,414 face images used to train our model. As for the evaluation, LFW [10], which contains 13,233 images with 5749 identities collected from the Web with large variations in pose, age, expression, illumination, and so forth, and YTF [3], a video dataset containing 3425 videos of 1595 different subjects downloaded from YouTube, were used. We considered the unsupervised protocol and followed the standard setting as described in [26]; in addition to the verification accuracy (Acc.), we used the ROC Receiver Operating Characteristic Curve to evaluatethe performance. We conducted the evaluation under the following setting: a cross-dataset validation, in which external data (CASIAWebFace) exclusive to LFW/YTF was used for training in order to show the generalization ability across different datasets. The datasets we used for validation were the LFW dataset in view #2, which has 6000 pairs, and the YTF face dataset, which has 5000 face pairs. As for the scene dictionary learning, we also exploited CASIAWebFace as the training dataset.

### 4.2. Training Process for the New Model

**Figure 10:**
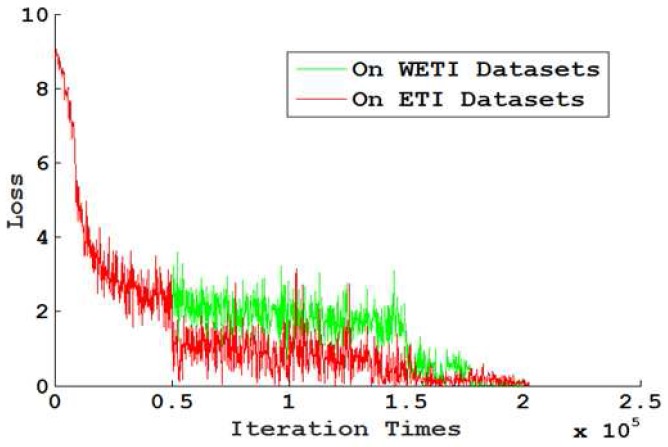
The loss on ETI datasets and WETI datasets with the same network model

To find the differences with enough training images (ETI: training datasets augmented using scene transformation) and without enough training images (WETI: using the raw data as the training data), 250,000 iterations were run on both the ETI and WETI datasets. The observed loss (error) is illustrated in Figure 10. The green curve indicates the loss convergence speed for WETI and the red indicates that for ETI. One can observe that the convergence speed for ETI was quite fast. As discussed in the above section, Table 2 illustrates the feasible hyperparameters for our proposed model.

### 4.3. The Semantic Features’ Extraction

**Figure 11:**
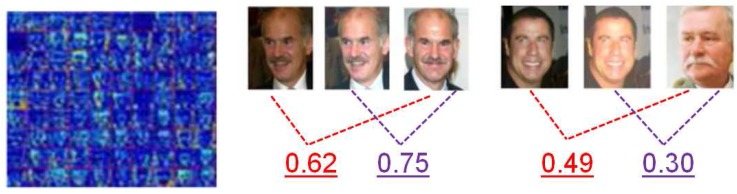
The extracted semantic feature and their responses to individuals, the value stands for cosine similarity

As mentioned in the model description section, the semantic features are extracted after the CRF–RNN process. Essentially speaking, CRF–RNN is exploited to build a relationship between a scene and face by extracting the face regions from the scene backgrounds in which they co-exist (refer to Figure 6). Then the proposed CNN model can express the face feature with scene information in a semantic way. With the benefit of the representations from intermediate layers, we can turn any face image into a vector containing important scene attributes of the face. As shown in Figure 11, it is indicated that the semantic feature distribution for the same person tends to be very close (middle); on the contrary, the semantic feature distribution for different people varies considerably, as shown in Figure 11 (right), although the faces are transferred to the same scene level.

### 4.4. Distribution of Semantic Features

**Figure 12:**
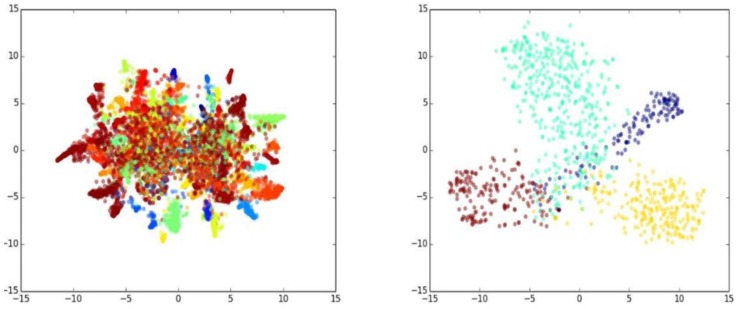
The features extracted by VGGFace.

We randomly selected 10,000 images from the CASIAWebFace dataset and then extracted their features using both the VGGFace model [27] and our proposed model. Because it is difficult to visualize the distributions of extracted features directly, *tSNE* [28] was usedfor reducing the extracted features’ dimension from a high dimension (10,575) to a low dimension (only 2). In this way, the differences in the distribution could be projected onto a two-dimensional plane. As shown in Figure 12, the same colors represent the same individual, and different colors represent different people. According to Figure 12 (left), the VGGFace model had a nearly circular feature distribution; that is, the extracted features of each category tended to gather together in a relatively small radius.

As we can see, about 50% of the categories gathered in a compact form, while the rest were scattered (see Figure 12 (left)). Zooming in on the details, there was extensive overlapping among the features from the selected four individuals, as observed in Figure 12 (right).

**Figure 13:**
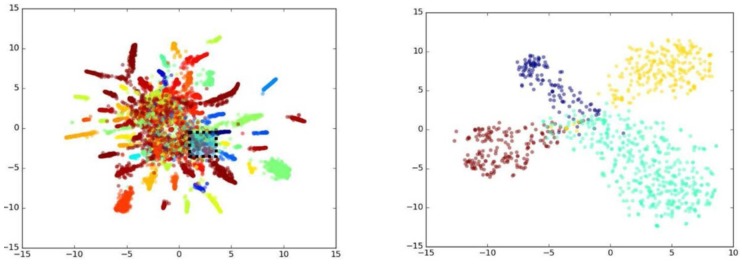
The features extracted by our method.

Next, we look at those features extracted by our proposed model. As we see in Figure 13 (left), the distribution of respective individuals looked more like a strip. It is also observed that about 80% of the categories were dispersed with a relatively larger spacing. Compared to the zoomed-in distribution property shown in Figure 13 (right), Figure 13 (right) illustrates less overlapping, and the distribution for inner classes was much more uniform along certain directions than that the VGGFace model produced.

### 4.5. Comparison with Other Models

**Figure 14:**
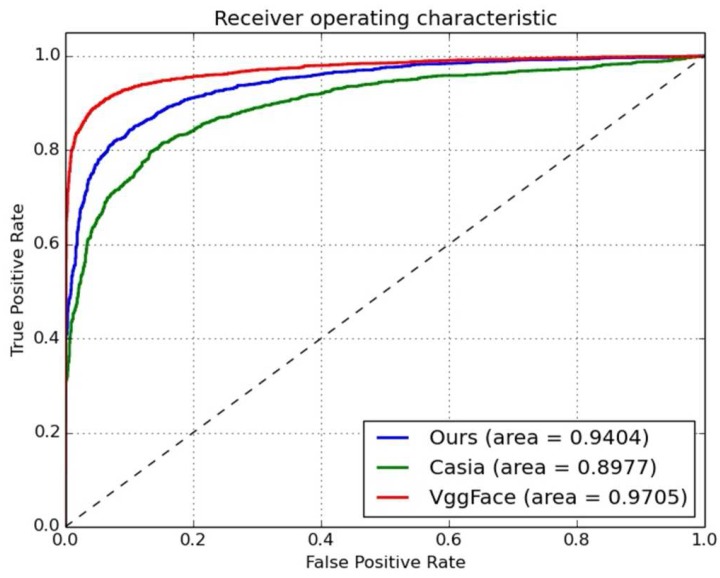
ROCs on LFW dataset with the unsupervised protocol.

In order to validate the performance of the proposed model, an unsupervised protocol [10] was used on both the LFW and YTF datasets. The reason we chose this protocol for comparison was that a strict generalization ability is necessary for the face-verification task in an open scene. Firstly, we compare the Casia [25] and VGGFace [27] models with our model. For the training step, all the models were trained on the CASIAWebFace dataset, and then these three models were validated on the YTF dataset. For the verification step, the proposed model needs the face pair to be transformed into the same scene (see Figure 9). The ROC curves for the three models on the unsupervised protocol were plotted, as shown in Figure 14. One can see that the performance of our proposed model on the LFW dataset was better than that of the Casia [25] model (Area Under Curve (AUC) gap of 0.0427), but the AUC of the VGGFace model was 0.0301, which was slightly better than for our model. However, the VGGFace model has an unrestricted protocol, and a much larger dataset for training is inevitable.

**Figure 15:**
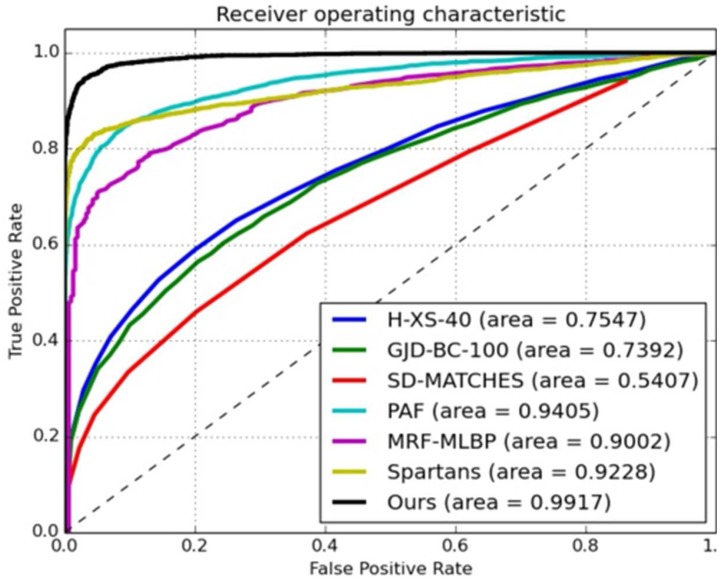
ROCs with different unsupervised models on LFW dataset.

**Figure 16:**
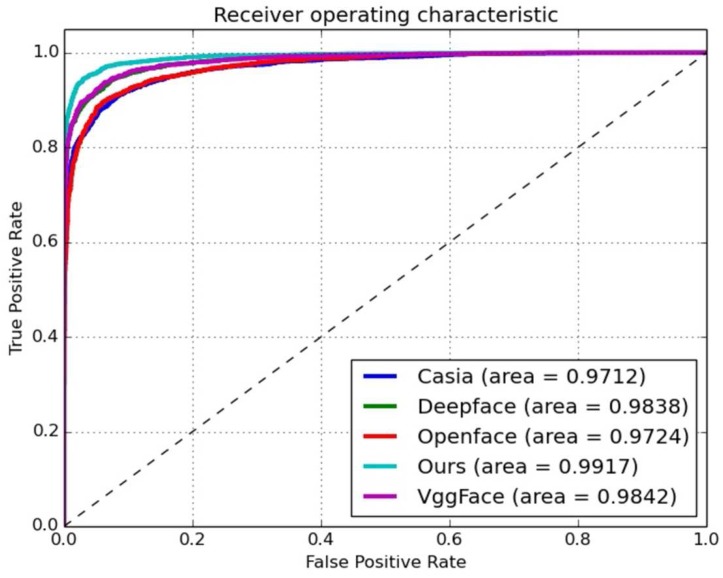
ROCs with unsupervised verification protocol on YTF dataset for some up-to-date models.

Secondly, we compared the AUC with the top 10 of the leading board [9,25,29,30] for the face-verification task on the LFW dataset (see Figure 15). We also compared several up-to-date leading models, such as Casia [25], DeepFace [18], OpenFace [31], and VGGFace [27], in an unsupervised protocol for the YTF dataset (see Figure 16). As observed in Figure 15 and Figure 16, our proposed model performed the best, and its AUC was 0.0075-fold higher than that of the VGGFace model.

**Figure 17:**
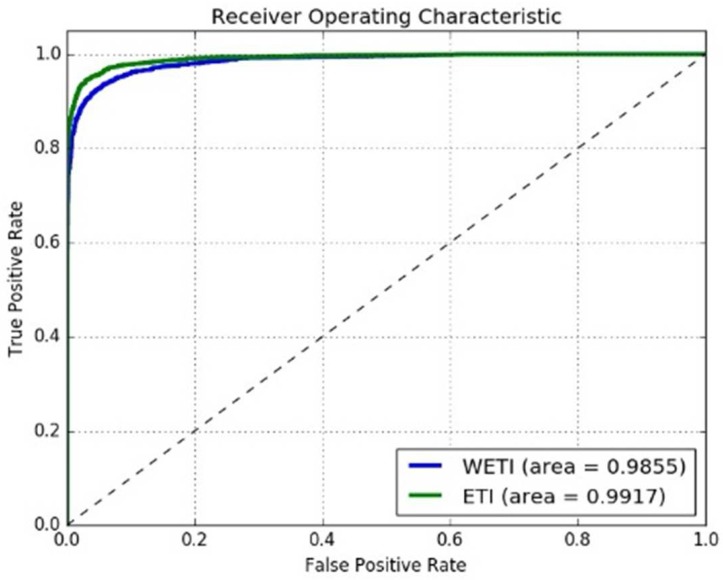
ROCs with unsupervised verification protocol on LFW dataset for without enough training images (WETI) and enough training images (ETI).

Thirdly, we summarize various protocols, training datasets, and networks in Table 3. Some performances listed in Table 3 are from the related publications. Here, it is shown that under the unsupervised protocol, our model achieved the best result on both the LFW and YTF datasets. In order to see the benefits much more clearly, we also compared the performances between ETI and WETI, which illustrated that ETI could gain an advantage of about 0.0062 over WETI on the LFW dataset, as shown in Figure 17.

## 5. Conclusions and Discussions

**Figure 18:**
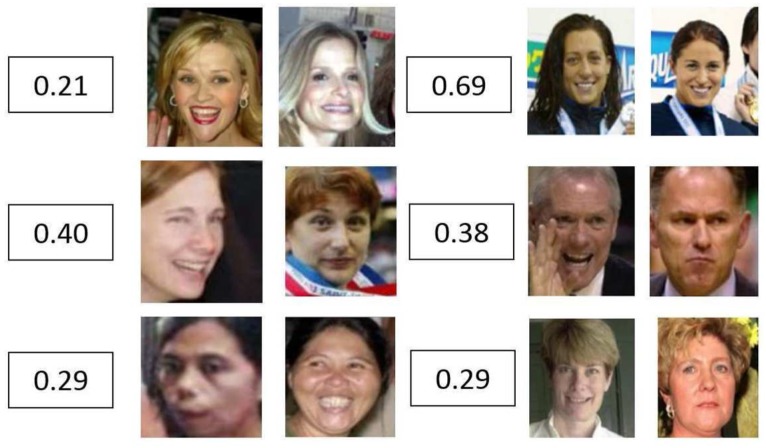
The face pairs in LFW were identified with great difficulty by other methods but could be distinguished between easily by our method.

In this paper, a new deep learning model is proposed for face verification and is essentially used to solve the small number of training samples requested by current deep learning networks. The main idea is to use scene transfer learning to generate more images for validation. The proposed model was evaluated from multiple perspectives. With the unsupervised protocol, our model performed better than the existing leading algorithms on the LFW dataset. According to Figure 16, its performance was at least 0.7% higher than that of other models under an unsupervised verification protocol. As illustrated by the ROCs, the VGGFace model slightly outperformed our proposed model on the YTF dataset; however, our model performed much better than the VGGFace model on the LFW dataset. The key point is that our model has much greater generalization capability, as it can deduce many more scenes for the training dataset. As observed in Table 3, it not only lists the performance and protocol, but also the training data size and network used by each model. As we can see, although our model was superior to DeepFace, with AUCs greater than those of LFW and YTF by 3.9% and 2.9%, respectively, the size of the training dataset was just 1/8 that of DeepFace. That is to say, we required much less data for our proposed model to train a better CNN model for the face-verification task. Even with the similar training data size (0.5 Million), in comparison with the CASIAWebFace model on the supervised protocol, our model achieved better results than LFW and YTF by 1.44% and 2.06%, respectively. The proposed model has only a relatively simple network structure. In contrast, the Baidu model uses 10 networks and had only about a 0.6% improvement with the **unrestricted protocol**. Although our model has only a simple network, it still achieved a steady performance for both LFW and YTF with an **unsupervised protocol**. The DeepID3 model has a much more complicated network (consisting of 200 models), but it achieved only 0.3% higher than the proposed model for LFW, even with a supervised protocol. The proposed model also outperformed the DeepID3 model by 0.9% for YTF with an unsupervised protocol. Hence, we can draw a conclusion that the proposed model can achieve a better performance than the state-of-the-art models with a relatively small amount of training data. As for the dataset LFW, there were several face pairs judged with great difficulty by the other models that could be distinguished between by the newly proposed model. As shown in Figure 18, the numbers stand for the cosine distance between a given face pair. When we set the threshold τ equal to 0.50, these face pairs could then be identified with less difficulty.

**Figure 19:**
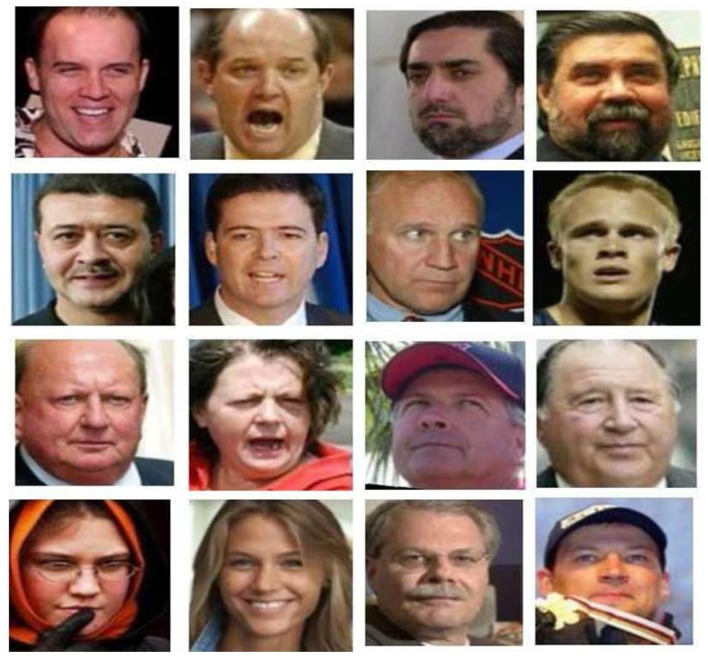
The pairs falsely accepted by the proposed model.

**Figure 20:**
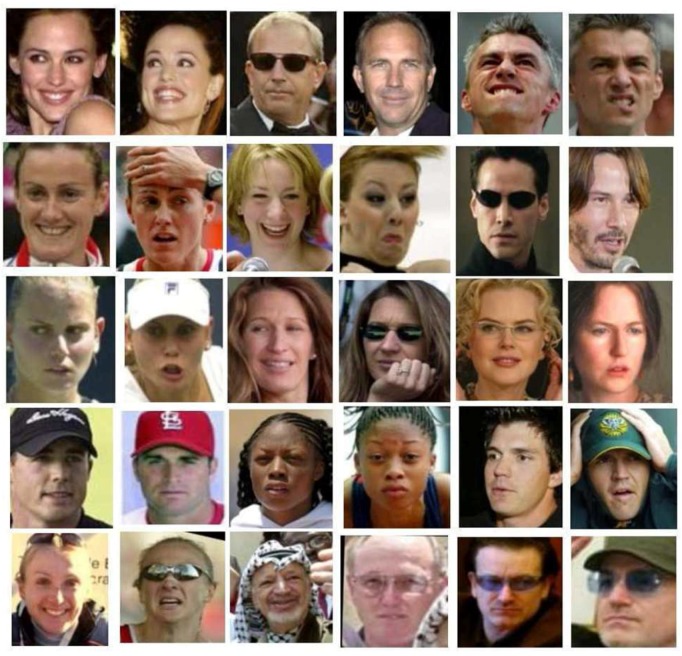
The pairs falsely rejected by the proposed model.

However, there were still some face pairs that our model failed to verify properly. Figure 19 shows those pairs that were falsely accepted by our proposed model, and Figure 20 illustrates those pairs that were falsely rejected by the proposed model.

As we can see, the key reason for the failure of our model was likely that the face images were subject to facial expressions, shelter, and other factors. Our next work will aim for profound research on the facial expression scene and make our model capable of transforming all facial expression scenes into uniform scenes.

## Figures and Tables

**Table 1:** Symbols and notation.

Symbol	Notation
s = ($s_{i}$, $s_{j}$)	Scene; number is unknown in advance
$\phi_{mix},$ $\phi_{pure}$	Two feature spaces, the pure and the mixed space
C	Category
p(,)	PDF (probability density function)
$I_{mix}$	The overlapping image space
θ	The statistics (μ; Σ)
I(u,v)	The pixel value at position u,v
$\pi_{k}$	The PDF of the *k*th scene
i,j,k	The image, category, and scene orders
$\phi_{x}$	Features extracted from the image

**Table 2:** Convolutional neural network (CNN) models and the parameters.

Name	Type	Stride	Output	#P
Conv11	Conv	(3, 3, 1)	(100, 100, 32)	280
Conv12	Conv	(3, 3, 1)	(100, 100, 64)	18,000
Pool1	Maxpooling	(2, 2, 2)	(50, 50, 64)	
Conv21	Conv	(3, 3, 1)	(50, 50, 64)	36,000
Conv22	Conv	(3, 3, 1)	(50, 50, 128)	72,000
Pool2	Maxpooling	(2, 2, 2)	(25, 25, 128)	
Conv31	Conv	(3, 3, 1)	(25, 25, 96)	108,000
Conv32	Conv	(3, 3, 1)	(25, 25, 192)	162,000
Pool3	Maxpooling	(2, 2, 2)	(13, 13, 192)	
Conv41	Conv	(3, 3, 1)	(13, 13, 128)	216,000
Conv42	Conv	(3, 3, 1)	(13, 13, 256)	288,000
Pool4	Maxpooling	(2, 2, 2)	(7, 7, 256)	
Conv51	Conv	(3, 3, 1)	(7, 7, 160)	360,000
Conv52	Conv	(3, 3, 1)	(7, 7, 320)	450,000
Pool5	AVGpooling	(7, 7, 1)	(1, 1, 320)	
Dropout	Dropout		(1, 1, 320)	3,305,000
Fc6	Fullyconnect		10,575	
Cost1	Softmax		10,575	
KL	Generate		10,575	2000
Total				5,017,000

**Table 3:** Performance on LFW and YTF databases.

Method	LFW	YTF	Protocol	Images	Networks
CNN-3DMM estimation [32]	92.35%	88.80%	Unrestricted	0.5 M	1
Casia [25]	97.73%	92.24%	Unrestricted	1.0 M	1
Pose/shape/expression augmentation [33]	98.07%	N/A	Unrestricted	2.5 M	1
VGGFace [27]	98.95%	97.30%	Unrestricted	2.6 M	1
Discriminative [34]	99.28%	94.90%	Unrestricted	0.7 M	1
SphereFace [35]	99.42%	95.00%	Unrestricted	0.5 M	1
DeepID [1:3] [19]	99.53%	93.20%	Unrestricted	0.3 M	200
CCL with AAM [36]	99.58%	95.28%	Unrestricted	0.5 M	1
Facenet [17]	99.63%	99.63%	Unrestricted	200 M	1
GTNN [37]	99.65%	N/A	Unrestricted	6.2 M	2
Baidu [16]	99.77%	N/A	Unrestricted	1.3 M	10
LBPNet [38]	94.04%	N/A	Unsupervised	0.5 M	1
Deepface [18]	95.20%	91.40%	Unsupervised	4 M	1
Casia [25]	97.30%	90.60%	Unsupervised	0.5 M	1
MRF-FUSION-CGKDA [29]	98.94%	93.20%	Unsupervised	0.5 M	5
AM-Softmax w/o FN [39]	99.12%	N/A	Unsupervised	0.5 M	1
Ours	99.2%	94.30%	Unsupervised	0.5 M	1

## Appendix A. Scene Dictionary Learning

**Algorithm 1** Scene dictionary learning
**Input** *X* datasets of detected and aligned faces **Output** *S*: scene dictionary for all individuals Begin Pretrain a CNN model on the basis of *X* by exploiting the default hyperparameters. Randomly initialize a scene entry matrix *S* whose size is M×N (*M* is the number of people, and *N* is the maximum length of faces for a given person). **for all**$face_{i}$ in *X* **do** $X_{new}$ ⟵ *X* by CRF Extract CNN feature $\phi_{i}$ for $face_{i}$ **end for** **for** each $person_{i}$ in *X* **do** Initialize the value $s_{p}$ with first scene **for** each i∈[1,N] **do** **if** Malanobis($\phi_{i}$, $\phi_{j}$) ≤ϵ **then** $face_{j}$ ∈ $s_{i}$ **else** $face_{j}$ ∈ $s_{i+1}$ $s_{p}$ ⟵ $s_{p}$ ∪ $s_{i+1}$ **end if** **end for** *S*⟵$s_{p}$ **end for** return *S* End

## Appendix B. Scene Inference and Model Training

**Algorithm 2** Scene inference and model training
Input: *X* datasets of detected and aligned faces; *S*: scene dictionary for all individuals Output: Enlarged datasets $X_{new}$ and a new trained CNN model 2: Begin 4: Sort the elements in *S* 6: **for** each j∈[1,S.length] **do** 8: Express the extracted faces’ features with a mixture Gaussian distribution p(f,s) 10: **for** each k∈[1,K] **do** 12: Determine $\pi_{k}$ of $S_{k}$ 14: Estimate $\hat{\mu }$ and $\hat{\sum }$ 16: **for** each person n∈[1,N] **do** 18: $\ln p(x_{n}|\pi ,\mu ,\sum )$ 20: = $L(\gamma ,x_{n},\theta )$ + $KL(q\left(\pi ,s|\eta \right)||p\left(\pi ,s|x_{n},\theta \right))$ 22: $x_{s_{new}}$ ← $\ln p(x_{n}|\theta )$ 24: update $X_{new}$ 26: **end for** 28: **end for** 30: **end for** 32: //EM iteration 34: **for all**t=1 to iter < *T* (time of total iterations) **do** 36: **First**, fix $E_{scene}$ to learn the CNN network 38: Extract features: 40: $\left(f_{x},f_{y}\right)=CNN\left(x_{i},x_{j}\right)$ “Conv”: $x_{j}^{l}=f_{cnn}\left(x_{i}^{l-1}\right)$ 48: “Pooling”: $x_{j}^{l}=f_{pooling}x\left(x_{j}^{l-1}\right)$ Backward Propagation: //Calculate the gradient of $E_{scene}$ $\delta =\frac{\partial E_{scene}}{\partial s_{k}}$ 58: //Transform back to the first layer of NN 60: $p\left(x_{t+1}\right)=p\left(x_{t}\right)+\delta \prod_{i=1}^{n}N(\Delta \mu_{k},\Delta \Sigma_{k})$ **end for** End
